# *De Novo* Mutation Rates in Sticklebacks

**DOI:** 10.1093/molbev/msad192

**Published:** 2023-08-30

**Authors:** Chaowei Zhang, Kerry Reid, Arthur F Sands, Antoine Fraimout, Mikkel Heide Schierup, Juha Merilä

**Affiliations:** Area of Ecology & Biodiversity, School of Biological Sciences, The University of Hong Kong, Hong Kong, Hong Kong SAR; Area of Ecology & Biodiversity, School of Biological Sciences, The University of Hong Kong, Hong Kong, Hong Kong SAR; Area of Ecology & Biodiversity, School of Biological Sciences, The University of Hong Kong, Hong Kong, Hong Kong SAR; Area of Ecology & Biodiversity, School of Biological Sciences, The University of Hong Kong, Hong Kong, Hong Kong SAR; Research Program in Organismal & Evolutionary Biology, Faculty Biological and Environmental Sciences, University of Helsinki, Helsinki, Finland; Bioinformatics Research Centre, Aarhus University, Aarhus, Denmark; Area of Ecology & Biodiversity, School of Biological Sciences, The University of Hong Kong, Hong Kong, Hong Kong SAR; Research Program in Organismal & Evolutionary Biology, Faculty Biological and Environmental Sciences, University of Helsinki, Helsinki, Finland

**Keywords:** mutation rate, divergence time, genetic diversity, germline mutation rate, ninespine stickleback

## Abstract

Mutation rate is a fundamental parameter in population genetics. Apart from being an important scaling parameter for demographic and phylogenetic inference, it allows one to understand at what rate new genetic diversity is generated and what the expected level of genetic diversity is in a population at equilibrium. However, except for well-established model organisms, accurate estimates of *de novo* mutation rates are available for a very limited number of organisms from the wild. We estimated mutation rates (*µ*) in two marine populations of the nine-spined stickleback (*Pungitius pungitius*) with the aid of several 2- and 3-generational family pedigrees, deep (>50×) whole-genome resequences and a high-quality reference genome. After stringent filtering, we discovered 308 germline mutations in 106 offspring translating to *µ* = 4.83 × 10^−9^ and *µ* = 4.29 × 10^−9^ per base per generation in the two populations, respectively. Up to 20% of the mutations were shared by full-sibs showing that the level of parental mosaicism was relatively high. Since the estimated *µ* was 3.1 times smaller than the commonly used substitution rate, recalibration with *µ* led to substantial increase in estimated divergence times between different stickleback species. Our estimates of the *de novo* mutation rate should provide a useful resource for research focused on fish population genetics and that of sticklebacks in particular.

## Introduction

Although much of the short-term evolution and adaptation is likely based on standing genetic variation (Barrett and Schluter 2008), new mutations are the ultimate source of genetic diversity. The rate at which new mutations arise is a key parameter in evolutionary biology and population genetics (Hartl and Clark 2007), but at the same time it is difficult to quantify as per-generation mutation rates are low (Lynch 2010). Traditionally, mutation rates (*µ*) have been estimated with the aid of locus-specific rates on the basis of phenotypes observed in crosses and pedigrees (e.g., Stadler 1930), mutation accumulation experiments (Mukai 1964) or inferred from sequence divergence among taxa (Kimura 1968). All these approaches make assumptions that are known to be frequently violated, and consequently, they can provide only gross approximations of *de novo* mutation (DNM) rates (Smeds et al. 2016).

**Fig. 1.**
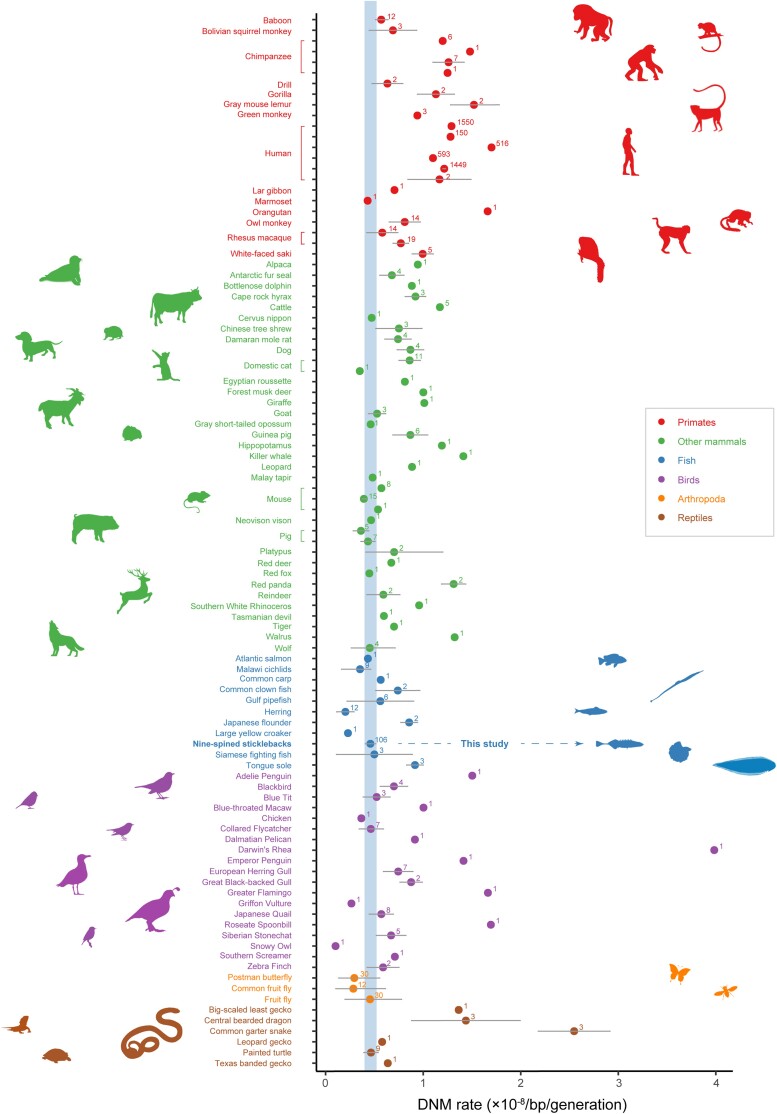
DNM rates to date. Per-site-per-generation DNM rate (10^−8^) estimates from studies which have used pedigree-based mutation rate estimation. The common names are sorted in alphabetical order for each taxonomic group. The number on each point indicates how many trios have been included in each estimate. Rates with no error bars do not have 95% confidence intervals available. For more details and references, please see supplementary table S1, Supplementary Material online. The blue shaded area indicates the confidence interval of DNM rate estimate for the nine-spined stickleback.

The drop in DNA-sequencing costs combined with improved variant calling methods have led to replacement of traditional approaches for mutation rate estimation with direct estimates obtained from DNA-sequence data (supplementary table S1, Supplementary Material online and fig. 1). However, direct estimation of DNM rates is not easy. Mutations are infrequent and each DNM has only 50% probability to be transmitted from a parent to offspring, and as such, a relatively large number of individuals from sequential generations need to be sequenced to have high detection probability. Even if enough DNMs can be confidently called, converting these to per generation (and year) mutation rates requires that the callable part of the genome (denominator of the rate estimate) is well estimated, which in turn requires a high-quality reference genome assembly (Besenbacher et al. 2015, 2019; Bergeron et al. 2022). In addition, to distinguish true DNMs from somatic mutations, controlling for false positive DNMs calls, each individual needs to be sequenced to high depth of coverage (Besenbacher et al. 2015; Bergeron et al. 2022). This means that mutation rate estimations are still costly for organisms with large genomes and not feasible for organisms lacking good quality reference genomes, against which sequenced reads can be confidently mapped. Furthermore, the mappable fraction of the genome should not be too small, and it should be well defined (Bergeron et al. 2022). Therefore, direct estimates of mutation rates are mostly available from model organisms with well-developed genomic resources and typically from unnatural captive or laboratory colonies (e.g., Keightley et al. 2014; Milholland et al. 2017; Lindsay et al. 2019; Wang et al. 2020; Bergeron et al. 2021). Recently, estimates have started to become available for a limited number of non-model organisms, such as cats, wolves, birds, and the duck-billed platypus (e.g., Keightley et al. 2015; Smeds et al. 2016; Martin et al. 2018; Koch et al. 2019; Yang et al. 2021), and most notably, for 68 vertebrate species (Bergeron et al. 2023). However, the pedigrees in most of these studies have been small, typically comprising a dozen of individuals or less (e.g., Smeds et al. 2016; Besenbacher et al. 2019; Koch et al. 2019).

The nine-spined stickleback (*Pungitius pungitius*) is a small teleost fish which has recently been subject to many population genomic, demographic and phylogenetic investigations (e.g., Guo et al. 2019; Natri et al. 2019; Varandhajan 2019; Yamasaki et al. 2020; Fang et al. 2021; Kemppainen et al. 2021; Feng et al. 2022; Kivikoski et al. 2022; Wang et al. 2022b), meaning that there is a community of researchers that would benefit from access to DNM rates in this species. This is because mutation rate estimates are key scaling parameters in many population genetic, demographic and phylogenetic inferences (e.g., Koch et al. 2019). Therefore, many-fold differences, for instance in estimates of migration rates, effective population sizes (*N_e_*), genetic diversity and divergence times among taxa, can ensue if inaccurate estimates of *µ* are used to derive them (Besenbacher et al. 2019; Koch et al. 2019; Tiley et al. 2020). In fact, studies of sticklebacks have so far resorted to using the substitution rates between three- (*Gasterosteus aculeatus*) and nine-spined stickleback (Guo et al. 2013) and an estimate of divergence time as proxy of per-year mutation rates (e.g., Liu et al. 2018; Ravinet et al. 2018; Varadharjan 2019; Yamasaki et al. 2020; Dahms et al. 2022; Feng et al. 2022).

Here, we aimed to obtain accurate estimates of DNM rates for outbred nine-spined sticklebacks using a high-quality reference genome assembly (Kivikoski et al. 2021) and deep (50×) sequencing of multigenerational pedigrees (2- and 3-generations) consisting of a total of 128 individuals from two marine populations (five families from each) separated by distance of over 300 km. In addition, we investigated where in the genome the DNMs occurred and whether they were associated with specific genomic features. Finally, we utilized these estimates to assess the consequences of using mutation rates instead of fossil calibration points and substitution rates to estimate divergence times among different stickleback species and lineages.

## Materials and Methods

### Sampling

**Fig. 2.**
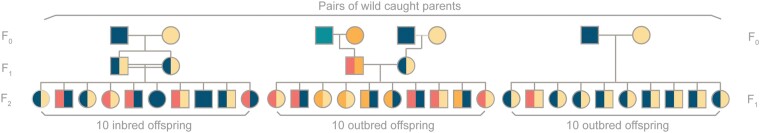
Pedigree types. Three types of pedigree structures used in this study: three-generation inbred line (left), three-generation outbred line (middle), and two-generation outbred line (right). Squares and circles represented males and females, respectively. The mutated alleles are shown in red and the normal wild types in other colours. Three possible scenarios have been presented here: 1) mutation transmitted from the F_1_ generation and shared between full-sibs (left); 2) mutation occurring in F_0_ germ cell and being transmitted to F_2_ offspring (middle); 3) non-shared mutation.

Twenty-two sexually mature male and female nine-spined sticklebacks, forming the F_0_ generation, were sampled with beach seine nets from May to June 2018 from Pori (POR; 61.591°N, 21.473°E) and Tvärminne (TVA; 59.833°N, 23.200°E) in Finland. Both localities are Baltic Sea coastal sites, and hence, the parental generation originated from outbred marine populations. The fish were transported to the aquaculture facility of Viikki Campus (University of Helsinki) and maintained in 17 °C in aerated aquaria until used in artificial fertilizations (for details of rearing conditions and procedures, see Fraimout et al. 2022). For each of the two marine populations, five 2- or 3-generational pedigrees were produced from the wild-caught F_0_ individuals by artificial crossing, where the last generation of each pedigree consisted of 10 full-sibs (fig. 2). Briefly, in vitro crosses were performed by squeezing eggs from females and combining them with minced testes dissected from males (euthanized with MS-222) in a Petri dish, where gametes were mixed gently to ensure fertilization. The resulting clutches were first reared in Petri dishes until they hatched and the fry started independent feeding. The F_1_ generation fish were reared for approximately 400 days (mean: 403.6 days), after which they were euthanized (using MS-222) or kept for breeding the F_2_ generation. In the case of the families where an F_2_ generation was produced, artificial crosses of F_1_ parents were performed as described above to produce F_2_ offspring, the latter of which were euthanized (with MS-222) and preserved in ethanol 2 days posthatching. All euthanized individuals (across generations) were stored in 95% ethanol to preserve DNA for extractions. Altogether, this study included 128 individual fish; 12 POR and 10 TVA F_0_ wild-caught specimens, 34 POR and 42 TVA F_1_ specimens and 20 POR and 10 TVA F_2_ specimens (Total n_POR_ = 66, n_TVA_ = 62). Hence, the number of trios (i.e., groups of two parents and their offspring) for POR and TVA were 54 and 52, respectively.

### DNA Extraction and Sequencing

Genomic DNA was extracted from fin clips using a modified salting-out protocol as described by Sunnucks and Hales (1996). The DNA purity of samples was evaluated with a NanoDrop spectrophotometer and the concentrations were quantified using Qubit dsDNA HS Assay Kit with Qubit^TM^ 4.0 (Invitrogen, CA, USA).

For TVA samples, the genomic DNA libraries were constructed with the NEBNext Ultra II FS DNA Library Prep Kit (Agilent, CA, USA). Thereafter, qPCR quantification of the sequencing libraries was conducted following the NovaSeq v1.5 protocol at the Biomedicum Functional Genomics Unit (FuGU) of the Helsinki Institute of Life Science (HiLIFE) and Biocenter Finland (BF) research infrastructure. DNA samples (1 µg) for each individual of the POR population were sent to Beijing Genomics Institute (BGI) for PCR-free library construction using their proprietary DNBseq platform for NGS sequencing. All samples were whole-genome sequenced to 50× target coverage.

### Read Mapping and Variant Calling

The paired-end data were processed following Feng et al. (2022). In brief, the raw reads were mapped to the most recent available nine-spined stickleback reference genome (version 7, GCA_902500615.3, Kivikoski et al. 2021) using the Burrows–Wheeler Aligner (BWA) with mem option (v0.7.17; Li 2013). Aligned reads were then sorted and indexed with mate coordinates flagged through SAMtools v1.10 (Li et al. 2009). The duplicate reads were marked with PicardTools (v2.18; http://picard.sourceforge.net). Base-quality score recalibration (BQSR) was also performed in GATK (v4.2.2.0; Van der Auwera and O'Connor 2020) using hard filtered SNPs and indels.

Following the best practices workflow of GATK (Poplin et al. 2017), the nucleotide variants were called using HaplotypeCaller in ERC mode, with several annotations being added (e.g., “MappingQuality”, “FisherStrand”, etc.) for downstream filtrations. The per-sample VCF files were then jointly genotyped by the CombineGVCFs and GenotypeGVCFs modules for each population and for each parent–offspring trio.

### Pedigree Examination

To confirm genetic relationships (e.g., parent–offspring) and structure of the pedigrees, several analyses were performed before germline mutation identification (where sex chromosome [LG12] and unassigned contigs were excluded). We firstly estimated the probabilities of identity-by-descent for each pedigree with PLINK (v1.90; Chang et al. 2015). The Z_0_:Z_1_:Z_2_ (probabilities of sharing no, one, and two alleles) for parent–offspring relationship should be close to 0:1:0, whereas that for full-sibs should be close to 0.25:0.5:0.25. We then also performed parentage analyses to check if the paired parents could be correctly assigned back to their offspring in FRANz (v1.9.999; Riester et al. 2009). The 012 matrices were generated in VCFtools (v0.1.16; Danecek et al. 2011) by allowing 10% missing genotype data and a minor allele frequency of 0.01. Finally, principal component analyses (PCA) for all individuals were performed to check that the individuals from the same pedigree would cluster together in PCA plots. The PCA plots were generated with ANGSD and PCANGSD (v0.939; Korneliussen et al. 2014). These checks confirmed that genetic relationships among the sequenced individuals were as assumed.

### Identifying the Candidate De Novo Mutations

For each parent–offspring trio (n = 106), the variants in each trio VCF file were filtered to a subset of single nucleotide variants (SNVs) by BCFtools (v1.10; Danecek et al. 2021) based on the Mendelian violation (fig. 2): we considered an offspring heterozygous variant (0/1) to be a DNM when their parents were both homozygotes for either reference (0/0) or alternative allele (1/1). A series of site filters and individual filters were then applied to these SNVs following the “Mutationathon” guidelines (Bergeron et al. 2022) which included:

- *Site filtering.* Following GATK best practice pipeline (Poplin et al. 2017), hard filtering was applied to all individuals to remove the low-quality positions with the following parameters: quality by depth (QD) < 2.0, mapping quality (MQ) < 40.0, Fisher's exact test on strand bias (FS) > 60.0, strand odds ratio (SOR) > 3.0, mapping quality rank sum test (MQRankSum) < −12.5, and read position rank sum test (ReadPosRankSum) < −8.0.
- *Individual filtering.* DNM candidates were filtered based on the following criteria to eliminate the false positives: 1) sequencing depth (DP ≤ 20 and DP ≥ 100) and genotyping quality (GQ ≤ 80) for both parents and their offspring were examined to exclude genotyping errors or read misalignments in regions of high complexity; 2) an allelic depth filter (AD1 > 0 for 0/0 or AD0 > 0 for 1/1) was applied for the two parents to ensure they are real homozygotes; 3) filters of allelic balance (AB < 0.3 and AB > 0.7) and sequencing depth (DP < 0.5DP_trio_ and DP > 2DP_trio_) were applied for offspring to confirm they are true heterozygotes; 4) an inspection to remove DNM candidates within 5 bp away from any indels to avoid any uncertainties brought by the realignment step; 5) removal of DNM candidates that occurred repeatedly in multiple unrelated samples but keeping a separate record of those shared among full-sibs; 6) a specific examination of the clustered sites where more than one DNM candidates were observed within 100 bp, as adjacent mutations are expected to occur with low probability, and most observed clustered candidates are therefore false positives caused by realignment of regions of repeats.

### De Novo Mutation Rate Estimation

The per-site-per-generation mutation rate (*µ*) for each offspring was calculated as:


(1) $$\mu =\frac{n_{\mathrm{DNMcandidate}}\;\;\times \;\;(1\;-\;\mathrm{FDR})}{2\;\;\times \;\;\mathrm{CS}\;\;\times \;\;(1-\mathrm{FNR})}.$$

The number of callable genome sites (CS) was obtained by applying a sequencing depth (DP) filter on the read alignments (bam files) in a given trio. The sites in an offspring were counted as “callable” only if they were with more than half and less than double of the total DPs within its trio family (0.5DP_trio_ < DP_child_ < 2DP_trio_), as was done for the candidate DNMs.

Assuming the true heterozygotes (0/1) in each offspring were those where one parent carried 0/0 and another had 1/1, we estimated the false negative rate (FNR) for each offspring as the percentage of true heterozygotes that did not pass the above-mentioned AB filter (AB < 0.3 and AB > 0.7).


(2) $$\mathrm{FNR}=\;\frac{n_{\mathrm{true}\;\mathrm{heterozygotes}\;\mathrm{being}\;\mathrm{removed}\;\mathrm{by}\;\mathrm{AB}}}{n_{\mathrm{true}\;\mathrm{heterozygotes}}}.$$

False positive DNMs were further identified manually using visualization by IGV (Thorvaldsdóttir et al. 2013), where the bam files from each trio set were checked at the same time to ensure that the raw reads supported each genotype, and only those that were well-supported were retained. Finally, the candidate DNMs were removed if, firstly, both or one of the parents carried the same mutation as their offspring (supported by up to 10% or more raw reads in sum which went undetected by GATK when genotyping each parent separately) or, secondly, the offspring was incorrectly identified as a heterozygote based on poor mapping in the positions around to candidate DNM. The false discovery rate (FDR) was then calculated from:


(3) $$\mathrm{FDR}=\;\frac{n_{\mathrm{false}\;\mathrm{positives}\;\mathrm{identified}\;\mathrm{in}\;\mathrm{IGVtools}}}{n_{\mathrm{all}\;\mathrm{candidates}\;\mathrm{after}\;\mathrm{individual}\;\mathrm{filters}}}.$$

The mutation rates were estimated for the two populations separately, but as they were not significantly different (see Results), we also combined all families to single analysis to gain accuracy in our estimate of *µ*.

### Mutation Spectrum and Genomic Context Analyses

Although DNMs are usually distributed randomly throughout the genome, they typically show distinct frequencies in relation to which mutational type they belong to (Milholland et al. 2017; Sasani et al. 2019; Wang et al. 2020). For example, mutation rates are observed to be particularly elevated in CpG sites where more deamination of methylated cytosines appears to occur (Razin and Riggs 1980; Zemojtel et al. 2011; Kong et al. 2012; Milholland et al. 2017; Wang et al. 2022a). First, mutation spectra were analyzed based on alternative and reference alleles in vcf files. Secondly, DNMs were divided into transversions (Tv: A:T > C:G, A:T > T:A, C:G > A:T, and C:G > G:C) and transitions (Ts: A:T > G:C and C:G > T:A). Thirdly, CpG islands (CGIs) were predicted by applying the “twoBitToFa” program (http://genome.ucsc.edu/cgi-bin/hgTrackUi?g=cpgIslandExt, Miklem and Hillier forthcoming) to the reference genome following criteria outlined in Gardiner-Garden and Frommer (1987). Because DNMs did not occur in CGIs of every individual, the rates of DNMs in CGI and non-CGI regions were estimated applying a zero-inflated method:


(4) $$\mu =\frac{\mathrm{sum}(n_{\mathrm{DNMs}\;\mathrm{detected}\;\mathrm{from}\;\mathrm{each}\;\mathrm{offspring}})}{2\;\;\times \;\;\mathrm{sum}(\mathrm{region}\;\mathrm{lengt}h_{\mathrm{each}\;\mathrm{offspring}})}.$$

The rate was also estimated for each type of CGI (intragenic CGI, intergenic CGI, transcription start site CGI, or transcription termination site CGI) classified according to where the CGI was located.

Additionally, the DNMs were phased back to their parent-of-origins by applying POOHA (https://github.com/besenbacher/POOHA) for the purpose of examining potential parental bias in mutation rate. Finally, we annotated each mutation in relation to the genomic location (*viz*. within exon, intron or outside coding sequence) and mutation type (non-synonymous [NS] or synonymous [S]) according to annotations in the previously published assembly of nine-spined stickleback (Version 6; Varadhajan 2019; Varadhajan et al. 2019) and a liftover file to Version 7 (Rastas 2020; https://sourceforge.net/p/lep-anchor/code/ci/master/tree/liftover.awk).

### Phylogenetic Dating

To understand the impact of the estimated DNM rate on divergence time estimates, we reconstructed evolutionary relationships of Guo et al.'s (2019) dataset for RAD-seq data of stickleback lineages using the BEAST package (v.2.6.7; Bouckaert et al. 2019) with two different dating approaches. Herein, the input dataset consisted of 1,708 SNPs from 65 *Pungitius* individuals representing seven independent lineages, as well as of four *Gasterosteus* and two *Culaea* sticklebacks as outgroups. Specifically, we compared the divergence times of two phylogenies/evolutionary scenarios, selecting and setting different rate priors for dating: 1) our estimate of the DNM rate—where we converted the per generation estimates of *µ* to per million year by assuming a generation length of two years (De Faveri et al. 2014); and 2) the synonymous substitution rate (SSR) between three- and nine-spined sticklebacks (7.1× 10^−9^/bp/yr; Guo et al. 2013)—which has been widely applied in literature to date (see Introduction). Although the branch topology was not fixed in the two scenarios, the overall branch lengths would still be representative of how quickly the species diverged as mutation rate was the only prior in the analyses. For congruence in each scenario, input files were constructed in BEAUti (BEAST package) using an optimized relaxed clock approach and the Yule tree prior. Therein to limit error and obtain the most accurate phylogeny, four independent runs of 100 million generations (sampling every 10,000 generations) were conducted as implemented in BEAST. They were then combined using LogCombiner (BEAST package) with 10% burnin (as assessed by parameter convergence in Tracer v.1.7.2; Rambaut et al. 2018). Tracer was again used to ensure that combined log files’ effective sample size values were >200 in each scenario. TreeAnnotator (BEAST package) was then used for each scenario independently to summarize trees with no further burn-in for nodal support and date comparisons (supplementary fig. S4, Supplementary Material online). Finally, the focal phylogenies presented were plotted with DensiTree which visualizes the quantitative patterns across all trees (Bouckaert 2010).

## Results

### De Novo Mutation Rates in Nine-spined Sticklebacks

A total of 1.17 million autosomal (and pseudoautosomal) variants (an average of 11,007 variants per offspring) passed the Mendelian violation filter. Herein, 534 putative DNMs were detected in POR and TVA families excluding those shared among siblings. After visualization with the IGVtools, the number of unique DNMs were reduced to 167 and 141 for POR and TVA, respectively. The DNMs were widely dispersed throughout the genome (supplementary fig. S1, Supplementary Material online). Power of all individual filters are reported in supplementary table S2, Supplementary Material online and supplementary figure S2, Supplementary Material online, and no detectable batch effects were observed.

**Fig. 3.**
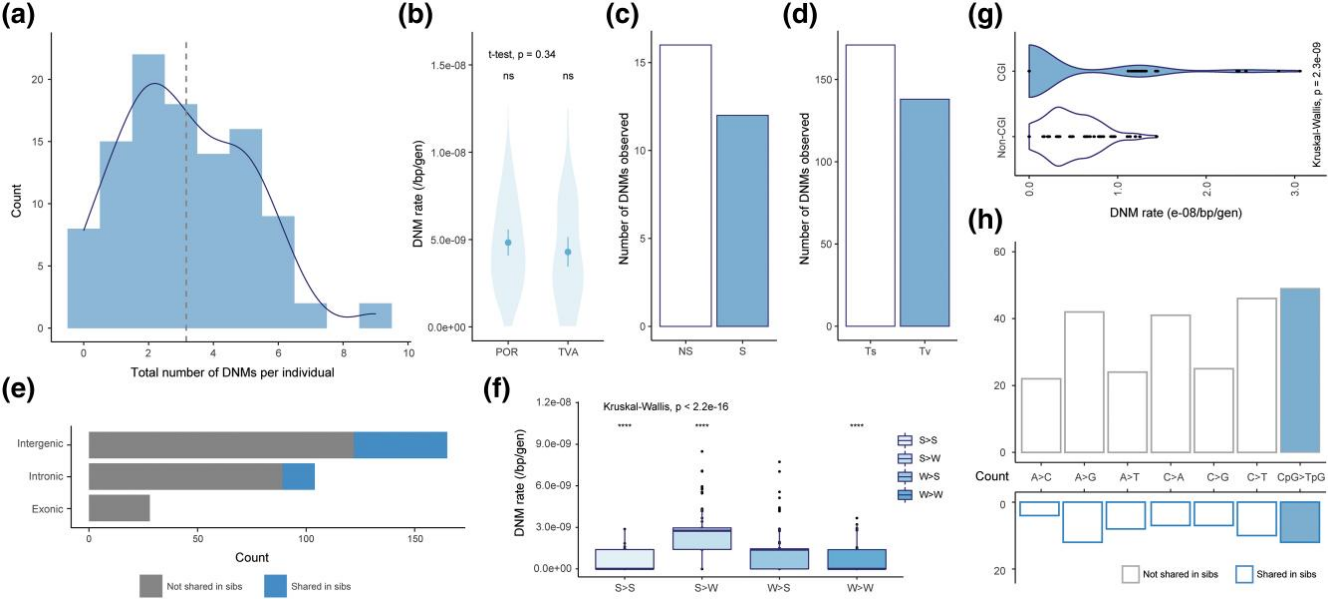
Mutation rates, types, and spectra. (*a*) Frequency distribution of DNMs detected per individual (dotted line is the mean = 3.16). (*b*) Mean DNM rates in POR (4.83 × 10^−9^ [95% CI: 4.09–5.56 × 10^−9^]) and TVA (4.29 × 10^−9^ [95% CI: 3.45–5.13 × 10^−9^]) populations (t_101.63_ = 0.96, *P* = 0.34). (*c*) Observed number of non-synonymous (NS) and synonymous (S) DNMs. (*d*) Observed number of transversion (Tv) vs. transition (Ts) mutations. (*e*) Number of DNMs located in intergenic, intronic, or exonic areas, categorized according to mutations being shared among full-sibs (blue) or not (gray). (*f*) DNM rate of strong-to-weak pairing type (S > W) mutations—note that this was significantly higher (Kruskal–Wallis test) than in the other types (S > S: C > G, S > W: C > A or C > T, W > S: A > C or A > G, W > W: A > T. The respective median DNM rates after corrected by FNR were: 0, 2.76, 1.39, 0 × 10^−9^/bp/generation). (*g*) Comparison of per-sample DNM rates in CpG island and non-CpG island regions. (*h*) Mutation spectrum of the detected DNMs separated according to if mutations were shared among siblings (below, blue border) or not (above, gray border).

Based on the read depths among trio families, the mean callable genome size was estimated to be 367.80 Mb, 86.78% of the entire genome without sex chromosomes (but including the pseudoautosomal region, >16.9 Mbp on LG12) and unassigned contigs. The average FNR was 5.81%, based on a test of 191,024 true heterozygotes per offspring. We inspected both the original and realigned (by choosing “-bamout” function) bam files for the variant calling step in IGVtools and found that the realignment procedure often led to disappearance and appearance of candidate DNMs. Thus, the manually curated FDR was 53.9% before and 21.9% after realignment in the GATK HaplotypeCaller. Following Bergeron et al. (2021), we eventually adopted the more conservative approach (the former one) which detected an average of 3.16 DNMs per individual (fig. 3*a*). All detected DNMs were mutated from the reference alleles (0/0 to 0/1 mutations). Combining all the statistics above, the final estimate of single-nucleotide germline mutation rate was 4.56 × 10^−9^/bp/generation (95% confidence interval [CI]: 4.01–5.12 × 10^−9^). This translates to a yearly DNM rate of 2.28 × 10^−9^/bp/year (CI: 2.01–2.56 × 10^−9^/bp/year) assuming a generation time of 2 years. There was no significant difference in DNM rate between the two populations (POR: 4.83 × 10^−9^, CI: 4.09–5.56 × 10^−9^ and TVA: 4.29 × 10^−9^, CI: 3.45–5.13 × 10^−9^; *t*-test: *t*_101.63_ = 0.96, *P* = 0.34; fig. 3*b*), the two sexes, pedigree types (inbred vs. outbred, fig. 2), or offspring generations (supplementary fig. S3*a*–*c*, Supplementary Material online). The rates of DNMs transmitted from F_1_ to F_2_ met with the expectation of binomial distribution, with a mean at 42.86% and CIs (33.5–55.2%) overlapping with the 50% expectation (χ^2^ = 0.02, df = 1, *P* = 0.89), and they were not statistically different between two pedigree types either (supplementary fig. S3*d*, Supplementary Material online).

### Characterization of Mutation Spectra

Among all unique DNMs, 55.2% were transitions (Ts) while 44.8% were transversions (Tv), showing a Ts:Tv ratio of 1.23 (χ^2^ = 66.24, df = 1, *P* = 4.0e−16; fig. 3*d*). The most common mutation type was C:G to T:A transition (116 out of the total 308), of which 52.6% were CpG > TpG mutations. We also observed a higher proportion of strong-to-weak pairing DNMs (S > W, C:G > A:T or C:G > T:A, 53.2%) with a median rate of 2.76 × 10^−9^/bp/generation. This DNM rate was significantly higher than the other types of substitutions in pairwise Wilcoxon test (vs. S > S: *P* < 2e−16; S > W: *P* = 4.1e−08; W > W: *P* = <2e−16; fig. 3*f*).

Of the DNMs residing on the annotated parts of the v6 genome assembly (Varandhajan 2019), 165 were within intergenic areas and 103 within introns, whereas 28 resided within gene coding sequences (CDS) and 12 in untranslated regions (UTRs, fig. 3*e*). There was no significant difference between the observed DNM frequencies and their expectation given the genomic coverage of each category (*viz.* intergenic, intronic, or exonic; χ^2^ = 5.25, df = 3, *P* = 0.155). Fourteen DNMs were found to be clustered, all of which were located outside exons. A total of 16 NS and 12 S exonic mutations were detected, among which 13 were CpG to TpG mutations (8 and 5 for NS and S DNMs respectively). Furthermore, only one exonic DNM was found at a splicing site which shifted the translation frame, potentially causing a loss-of-function (LOF) to the CDS. Except for this, no other LOF DNMs were detected including stop-codon variants.

Furthermore, 31 point mutations were observed within CGI, accounting for 10.1% of all DNMs detected. These mutations on CpG islands were mostly CpG > TpG substitutions with a 2.77-fold higher DNM rate than in a non-CpG context (6.35 vs. 2.39 × 10^−9^/bp/gen, table 1). A notably reduced frequency of CpG > TpG DNMs was observed inside the CGI compared to the frequency outside (16 vs. 50, χ^2^ = 11.82, df = 1, *P* = 9.66e−05), which was not seen for the non-CpG sites (table 1). In addition, CGI CpG sites exhibited higher rates in CDS than the other genomic regions (supplementary fig. S6*b*, Supplementary Material online), and more appeared to be exonic instead of intronic, which contrasted with the pattern observed in other parts of the genome as mentioned above, accounting for the non-CGI CpGs (supplementary fig. S6*a*, Supplementary Material online).

**Table 1.** The Number and Rate of DNMs Estimated by Nucleotide Types

	Average callable genome size	Number of DNM	Average rate of DNM (/bp/gen)
ALL	367,091,170	335	4.56E-09
CGI	41,466,245	31	3.53E-09
CpG	11,890,473	16	6.35E-09
Non-CpG	29,575,773	15	2.39E-09
Non-CGI	325,624,925	304	4.40E-09
CpG	12,766,207	50	1.85E-08
Non-CpG	312,858,718	254	3.83E-09

CGI, CpG Island.

In our dataset where the last generation of all pedigrees consisted of 10 full-sibs, 60 mutations, accounting for 19.5% of the total 308 DNMs, were carried by two or more siblings of the same parents which suggested that they had occurred during early germ cell divisions (parental mosaicism; Zlotogora 1998). These mosaic mutations only occurred in intergenic and intronic regions, but not on exons (fig. 3*e*). Also, we did not detect any significant differences in mutation spectrum between shared and non-shared DNMs (χ^2^ = 2.57, df = 6, *P* = 0.86), including the fraction of CpG > TpG DNMs (20.0% vs. 19.8%; fig. 3*h*) or CGI variants (χ^2^ = 3.00, df = 1, *P* = 0.08). Approximately 69% DNMs were assigned back to their parent-of-origin, where we observed significantly more CpG > TpG mutations in DNMs inherited from fathers than mothers (χ^2^ = 7.07, df = 1, *P* = 0.0078; supplementary fig. S5, Supplementary Material online). The percentage of paternal derived mutations was not significantly different in shared DNMs from those that were not shared (χ^2^ = 0.16, df = 1, *P* = 0.68). In total, ∼60% (CI: 52.5–68.0%) DNMs were found inherited from the paternal side, which translated to a male-to-female ratio (α) of 1.52. No significant difference was found in α between generations in the generalized linear model (F_(1,76)_ = 0.36, *P* = 0.552).

### Divergence Time Estimation With DNM Rates

**Fig. 4.**
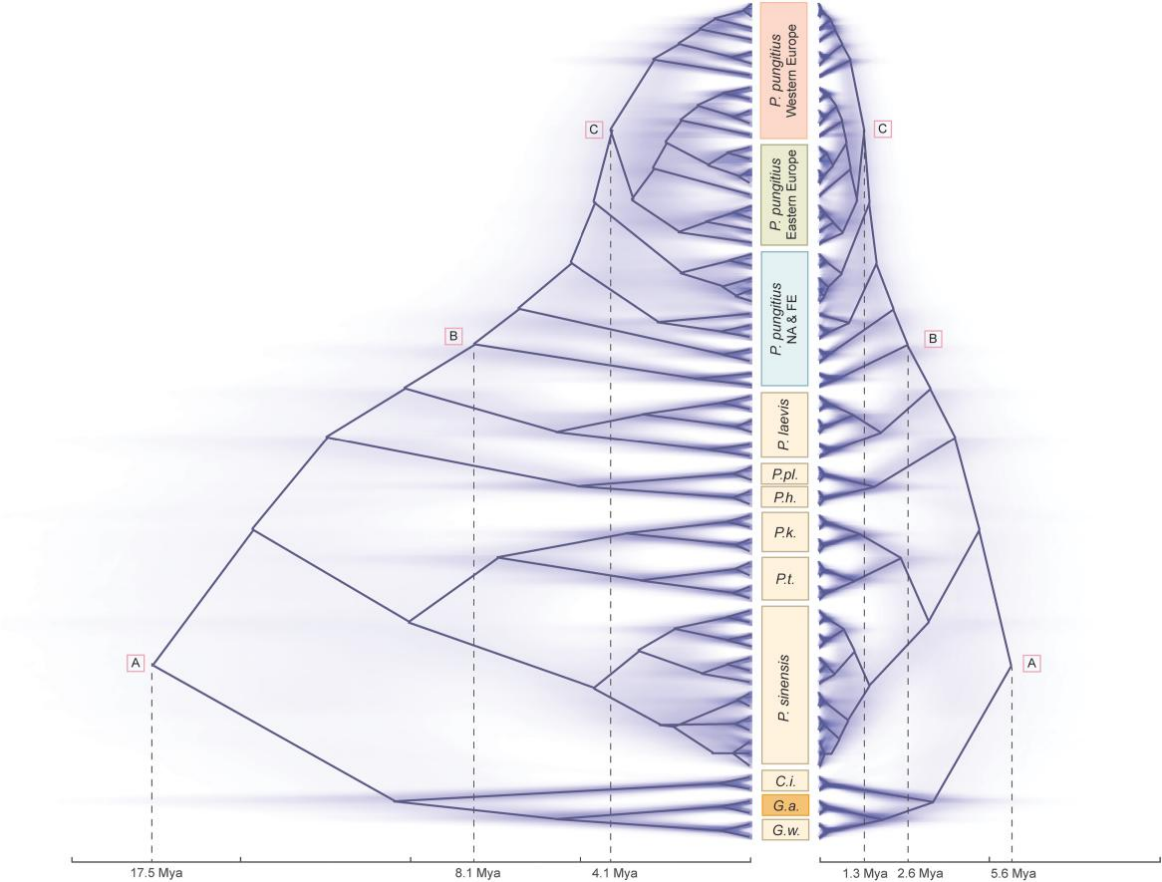
The DNM and substitution rate-based phylogenies of *Pungitius* sticklebacks. The DNM rate-based tree on left and substitution rate-based tree on right. Solid lines represent the summarized canal trees with maximum clade credibility scores, while the faint lines represent consensus trees for all topologies. (*P.pl.*, *P. platygaster*; *P.h.*, *P. hellenicus*; *P.k.*, *P. kaibarae*; *P.t.*, *P. tymensis*; *G.w.*, *G. wheatlandi*; *G.a.*, *G. aculeatus*; *C.i.*, *Culaea inconstans*; FE, Far Eastern lineage; NA, North American lineage).

Phylogenies, following the two dating approaches, generated in BEAST contained many well-supported nodes (supplementary fig. S4, Supplementary Material online). Although there was one instance of branch swapping between the phylogenies (Node D; supplementary fig. S4, Supplementary Material online), it is important to note that this node lacked significant support in both scenarios (PP ≥ 0.95) and otherwise the phylogenetic relationships were conserved. The dates of three nodes that most studies have focused on (i.e., A = divergence of *P. pungitius* and *G. aculeatus*; B = the MRCA for all *P. pungitius* lineages; C = divergence of eastern and western European lineages of *P. pungitius*; Guo et al. 2019; Feng et al. 2022) were shared and supported in both topological scenarios (fig. 4 and supplementary fig. S4, Supplementary Material online), though these show key differences in dates depending on the method applied: the date comparison of scenarios using Guo et al.'s (2013) SSR vs. our DNM rate showed the divergence time of node A shifted from 5.6 Mya (95% highest posterior density [HPD]: 3.3–8.1 Mya) to 17.5 Mya (95% HPD: 10.6–25.3 Mya), node B shifted from 2.6 Mya (95% HPD: 1.4–4.0 Mya) to 8.1 Mya (95% HPD: 4.4–12.5 Mya), and node C shifted from 1.3 Mya (95% HPD: 0.7–2.0 Mya) to 4.1 Mya (95% HPD: 2.2–6.3 Mya)—equating to on average 3.13× older dates across the three nodes based on our DNM rate (fig. 4).

## Discussion

Although mutation rate is a fundamentally important quantity in evolutionary biology and genetics, accurate estimates for vertebrates beyond primates are still rare (supplementary table S1, Supplementary Material online and fig. 1). Here, we have provided a pedigree-based germline mutation rate estimate for sticklebacks based on, by far, the largest number of trios that any non-human study has used (supplementary table S1, Supplementary Material online and fig. 1). The estimated mutation rate for sticklebacks (0.456 × 10^−8^/bp/generation) is much lower (2.2–14.9×) than the rates that have been applied in earlier studies of sticklebacks (1.42 × 10^−8^, Guo et al. 2013; 6.8 × 10^−8^, Roesti et al. 2015; 3.7 × 10^−8^, Liu et al. 2016; and 1 × 10^−8^/bp/generation, Liu et al. 2018). Not surprisingly, application of the new mutation rate estimate established herein, as a prior when dating divergence times among stickleback clades, pushed the estimated divergences back in time quite considerably (fig. 4 and supplementary fig. S4, Supplementary Material online).

To date, pedigree-based germline mutation rates have been estimated in 86 eukaryote species in 35 separate studies (supplementary table S1, Supplementary Material online and fig. 1). Most of these estimates come from studies of humans and primates (62.9%), followed by studies of other mammalian species (22.9%). Despite teleosts being the most species-diverse group of vertebrates (Venkatesh 2003), germline mutation rate estimates have only been estimated in 11 teleost fish species (supplementary table S1, Supplementary Material online, shaded in blue), including eight species recently reported by Bergeron et al. (2023). Our estimate for nine-spined sticklebacks resides in the middle of the other germline mutation rate estimates in fish (µ = 2–9.1 × 10^−9^/bp/generation, Feng et al. 2017, Malinsky et al. 2018, Bergeron et al. 2023, fig. 1 and supplementary table S1, Supplementary Material online) exhibiting the narrowest 95% CI. Overall, the 95% CI for 28 species in figure 1 overlap with our estimate, including 13 whose mean DNM rates lie exactly within this range (asterisked and underlined species in supplementary table S1, Supplementary Material online). However, given the rates are mostly estimated from data consisting of less than 10 trios, any generalizations about mutation rates in these species and their magnitude relative to other taxonomic groups requires additional pedigree-based estimates from larger datasets to become available.

Low temperatures have been suggested to influence mutation rates due to slower metabolic rates (Martin and Palumbi 1993). Feng et al. (2017) discussed this as a possible factor explaining the low mutation rate in the Atlantic herring. While the higher mutation rates of Lake Malawi cichlids living in warmer waters align with this explanation (Malinsky et al. 2018), the even higher mutation rate estimates for nine-spined sticklebacks herein contradict it. Namely, the nine-spined sticklebacks used in our study originate from the Baltic Sea where sticklebacks are exposed to the same thermal conditions and metabolic constraints as Atlantic herrings. Hence, the effect of environmental temperature on metabolic rates, and thereby to mutation rates, do not seem to be a likely explanation for low mutation rate in the Atlantic herring.

Parental age and gender are known to influence mutation rates in vertebrates. Our estimate of the mutation rate per generation could be a slight underestimate compared to the situation in the wild if older parents generate and transmit more mutations to their offspring (e.g., Kong et al. 2012; Wong et al. 2016; Jónsson et al. 2017; Wang et al. 2022a). This is because our lab reared parents (F_1_ individuals in 3-generation families) were probably younger than their wild-caught parents. However, since more wild-caught (n = 22) than F_1_ (n = 6) parents were included into the analysis, any bias due to age variation is unlikely to be large. In fact, mutation rates estimated from wild-caught and F_1_ parents did not differ (*t*_55.18_ = −1.09, *P* = 0.28; supplementary fig. S3*a*, Supplementary Material online). Furthermore, one should also note that per-year mutation rate estimates are subject to assumptions regarding the generation time used. For populations with overlapping generations, the generation time equals the mean age of parents (Hill 1979). Although we do not know the age of wild-caught parents, the use of published estimates of the age of reproductive Baltic Sea sticklebacks (De Faveri et al. 2014) should provide a good proxy of the generation time for this species.

Fathers are known to generate and transmit more mutations to their offspring in primates (e.g., Kong et al. 2012; Wong et al. 2016; Jónsson et al. 2017; Wang et al. 2020; Wu et al. 2020), mice (Lindsay et al. 2019), domestic cats (Wang et al. 2022a), and birds (Ellegren and Fridolfsson 1997; Bergeron et al. 2023). Sperm is also generally more methylated than eggs, especially on CpG islands (Rahbari et al. 2016; Milholland et al. 2017), thus more DNMs are expected to be inherited from fathers. Whether this applies also to fish is unclear—Bergeron et al. (2023) did not observe any strong sex bias in analyses of eight fish species. In this study, we observed a slight male bias in *µ* (α = 1.52). The lack of male bias in *µ* in earlier fish studies has been attributed to the fact that female fish produce hundreds of eggs which can increase the frequency of maternally transmitted mutations (Bergeron et al. 2023). It has been also suggested that as fish tend to be seasonal breeders, producing sperm over a limited period in early mating season rather than continuously as birds and mammals, this could explain the lack in male bias (Bergeron et al. 2023). However, a peculiarity of stickleback biology is their prolonged breeding season during which multiple clutches are produced (Wootton 1976, 1984). While this could explain the male bias in *µ* in this species, a strong test of sex-specific mutation rates in sticklebacks needs to wait for a larger sample size of aged adults.

Mutations can be shared among full-sibs if they occur postzygotically at very early stages of development of the parental germline. We discovered a fairly large proportion (∼20%) of shared mutations in nine-spined sticklebacks. Our estimate is higher than those in primates (human: 1.3%; Rahbari et al. 2016 and 3%; Sasani et al. 2019; apes: 3.5%; Bergeron et al. 2021), birds or reptiles (2.2% and 8.1%; Bergeron et al. 2023), but similar to a value estimated in mice (18%; Lindsay et al. 2019) and other fish (12%; Bergeron et al. 2023). However, a comparable estimate from the herring is much higher (50%), but this estimate is based on a very small sample size (4 parents and 12 offspring; Feng et al. 2017). Nevertheless, it appears as if parental mosaicism can be higher in fish than in other taxonomic groups.

One of the advantages of having direct estimates of germline mutation rates is that they allow one to probe long-term effective population sizes by substituting *µ* and nucleotide diversity (π) to solve effective population size (*N*_e_ = π/4*µ*, Watterson 1975). This gives an estimated long-term *N_e_* for *P. pungitius* in the range of approximately 160,851–262,645 individuals. These estimates are an order of magnitude larger than estimates in Feng et al. (2022) obtained with coalescent methods (*N_e_* ∼15,000–40,000). Yet, these numbers are likely to still be orders of magnitude lower than actual census population sizes of sticklebacks in the Baltic Sea. However, one must remember that the *N_e_* derived from the equation above refers to populations in mutation-drift equilibrium. In the case of Baltic Sea sticklebacks, the equilibrium assumption is likely to be violated due to post glacial population expansion and rampant introgression between divergent *P. pungitius* lineages (Feng et al. 2022). All these factors will influence π and thereby also the *N_e_*. In the same vein, the drift threshold *N_e_*'s obtained from the equation above would be overestimated if mutation rates over the last 1–2 Mya have been declining (Burridge et al. 2008).

Our analyses of mutation spectra in sticklebacks were largely congruent with those from mammalian studies (Pfeifer 2017; Koch et al. 2019). For example, we found over-representation of C > T transitions, more frequent weak-to-strong pairing mutations and random distribution of DNMs in intergenic, intronic, and exonic areas. We also observed a high proportion of CpG > TpG mutations (19.48%), falling in the range observed in other species (9–25%, Venn et al. 2014; Smeds et al. 2016; Thomas et al. 2018; Besenbacher et al. 2019; Campbell et al. 2021). However, our estimate of Ts:Tv ratio (1.23) is on the lower tail of the distribution across 151 trios in vertebrates (mean = 2.3; Bergeron et al. 2023), where herring (1.43; Feng et al. 2017), Atlantic salmon, tongue sole, and Japanese flounder (0.67, 1.13 and 1.50; Bergeron et al. 2023) are also located at. This is because the nine-spined sticklebacks exhibit more C > A mutations than most of the other species. Bergeron et al. (2023) has also observed a slightly different mutation spectrum in fish exhibiting less A > C but more C > A mutations.

Furthermore, DNM rates in the CpG islands in our data share a similar pattern as observed in humans, with CpGs more resistant to mutations inside the CGIs as compared to those outside of them (Gardiner-Garden and Frommer 1987) and mostly located within intragenic CGIs than the other types of CGIs (Francioli et al. 2015; Youk et al. 2020). This is likely due to a lower level of methylation within CGI than non-CGI regions (Illingworth et al. 2010), but CGIs on gene bodies are still hypermethylated (Youk et al. 2020). The gene *prdm9* specifies where recombination mediated double-stranded breaks occur (Cavassim et al. 2022). Recombination hotspots have been found to be mainly determined by the genomic methylation patterns in dogs, which have lost functional *prdm9*, and their CpG mutation rate correlates negatively with the recombination rate (Berglund et al. 2014). Nine-spined sticklebacks appear to have lost *prdm9* according to a nucleotide BLAST in NCBI (https://www.ncbi.nlm.nih.gov/) against the reference genome (GCA_902500615.3). Therefore, a detailed comparison of the localized DNM rates and the recombination hotspots in sticklebacks could be of future interest.

Mutation rates are important in calibrating molecular clocks, as well as in converting branch lengths of genealogies to units of time (Kimura 1968; Koch et al. 2019; Tiley et al. 2020). Hence, any uncertainty about mutation rates can directly propagate to distort demographic inferences, such as divergence times, effective population sizes, and migration rates among populations (e.g., Ségurel et al. 2014; Koch et al. 2019). Our results provide a case in point: by calibrating the *Pungitius* phylogeny with our direct estimate of per-year DNM rates had a dramatic effect on divergence times pushing them back millions of years from the recent estimates (Fang et al. 2021; fig. 4). It is also worth noting that the divergence time estimates based on our DNM rate aligned better with the fossil-record based dating (7 Mya for MRCA for genus *Pungitius* spp; Rawlinson and Bell 1982) and with phylogenies based on direct and indirect fossil dating (e.g., Guo et al. 2019). While this provides further confidence to believe that divergence time estimates using our de novo mutation rate estimate are closer to the truth than the substitution-based estimates, one should keep in mind that mutation rates may evolve over time and/or vary among different lineages (e.g., Pozi and Penna 2022). This variation would naturally influence estimated divergence times. In this perspective, further studies should seek to obtain mutation rate estimates from other members of the family Gasterosteidae.

While leveraging empirically estimated mutation rate in divergence time estimation has its advantages (Tiley et al. 2020), one has to remember that the estimated divergences need to be scaled to absolute time units using generation time. Hence, any errors or biases in applied generation time will progate with the divergence time estimates. Since there is considerable variation in life span (3–7 years) and likely also generation time (defined as average age of breeding parents in the population; Hill 1979) among different nine-spined stickleback populations (De Faveri et al. 2014), this is clearly a point of potential concern. However, since nothing is known about the generation times in other *Pungitius* species, it is difficult to know if this constitutes a problem for divergence time estimation and if so, how big. Whether the magnitude of this potential problem is anywhere close to the difference we observed when estimating divergence times with the synonymous substitution rate vs. mutation rate as the priors requires better understanding of basic biology of different stickleback species.

Finally, although DNM rates are known to be higher in mitochondrial than in the autosomal genome (Nabholz et al. 2008; Xu et al. 2012; Lawless, et al. 2020), we did not detect any mitochondrial mutations in our data. The reason for this is likely to be trivial: assuming a mutation rate of 1.67 × 10^−8^/bp/year (an average value of examples in Burridge et al. 2008) and given that the size of mitogenome is quite small, with only 16,720 bp for *P. pungitius* (Guo et al. 2016), one would need to survey at least 895 trios to find one mutation in mtDNA. Hence, estimation of mitochondrial mutation rate would require an entirely different sequencing strategy to the one employed in the present study.

In conclusion, the results provide the first and accurate estimate of *µ* for a popular stickleback model system in evolutionary biology. They further show that application of this estimate on divergence time calibration among different stickleback clades pushes back the earlier estimates of divergence times among different lineages, highlighting its utility in phylogenetic and demographic inference. Compared to mutation rate estimates in other eukaryotes and teleost fishes, the stickleback estimate falls into the middle range being very similar to that of Atlantic salmon. As the estimates in this study came from outbred marine populations, future estimates of *µ* from isolated freshwater populations, as well as from closely related species, could provide insights on factors contributing to the evolution of mutation rates.

## Supplementary Material

msad192_Supplementary_Data

## Data Availability

The raw whole-genome sequencing data and the VCF files can be obtained from the European Nucleotide Archive (ENA; https://www.ebi.ac.uk/ena) under accession code PRJEB60682. The nexus alignment files applied in phylogenetic analyses were compiled from Guo et al. (2019) and the.xml control files are available as supplementary files. The annotation file of the reference genome (Version 6) was obtained from Varadharajan *el al*. (2019; https://doi.org/10.6084/m9.figshare.10565507.v1) and the liftover file to Version 7 was published by Rastas (2020; https://sourceforge.net/p/lep-anchor/code/ci/master/tree/liftover.awk). The codes used in the analyses are available on GitHub (https://github.com/zcharlene/dnmrate9spinedmarine).
