# Supplementary material for: De Novo Mutation Rates in Sticklebacks
Source: Mol Biol Evol. 2023 Aug 30;40(9):msad192. doi: 10.1093/molbev/msad192 (PMC10503787; doi:10.1093/molbev/msad192)
Supplement: msad192_Supplementary_Data [file msad192_supplementary_data.zip › Supplementary- De novo mutation rates in sticklebacks.pdf]

Supplementary materials

Supplementary Fig. 1. Detected de novo mutations (DNMs) mapped on *P. pungitius* chromosomes. Vertical lines depict DNMs and they are coloured according to their genomic location. Included are autosomal linkage groups and pseudoautosomal part (non-grey region) of the linkage group 12 (sex chromosomes).

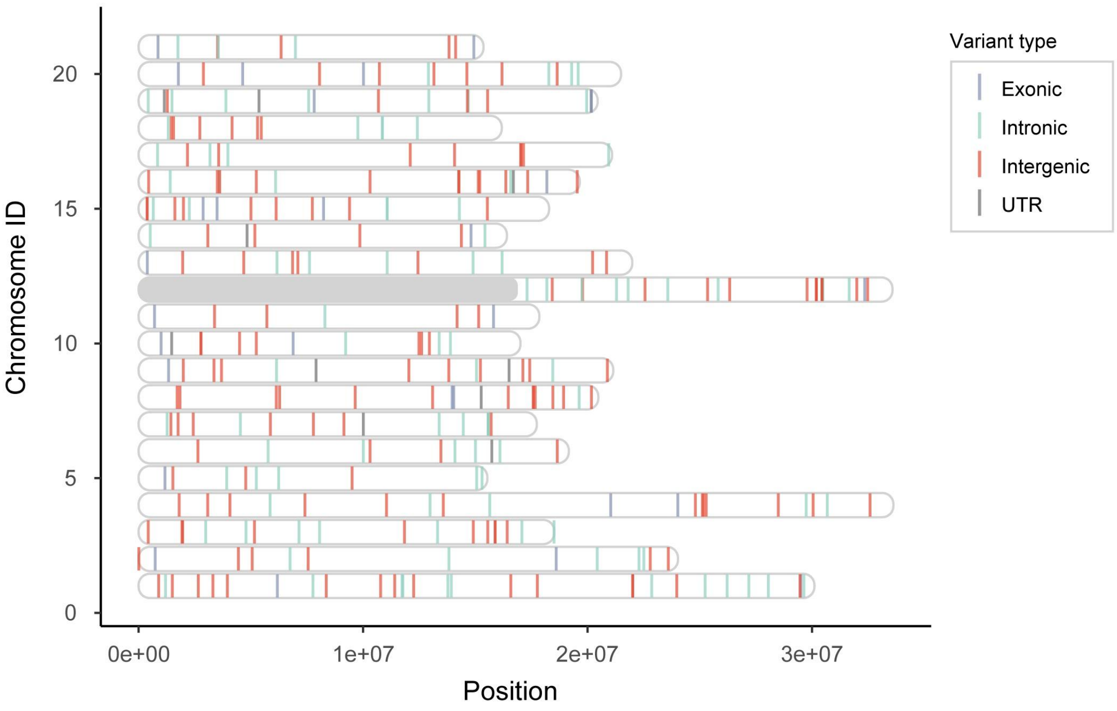

Supplementary Fig. 2. Power of filters across different families. Number of *de novo* mutation candidates removed by a) all filters after site filtering and Mendelian violation; b) a zoomed in look at the last four filters applied. TVA = Tvärminne family (n = 5), POR = Pori family (n = 4).

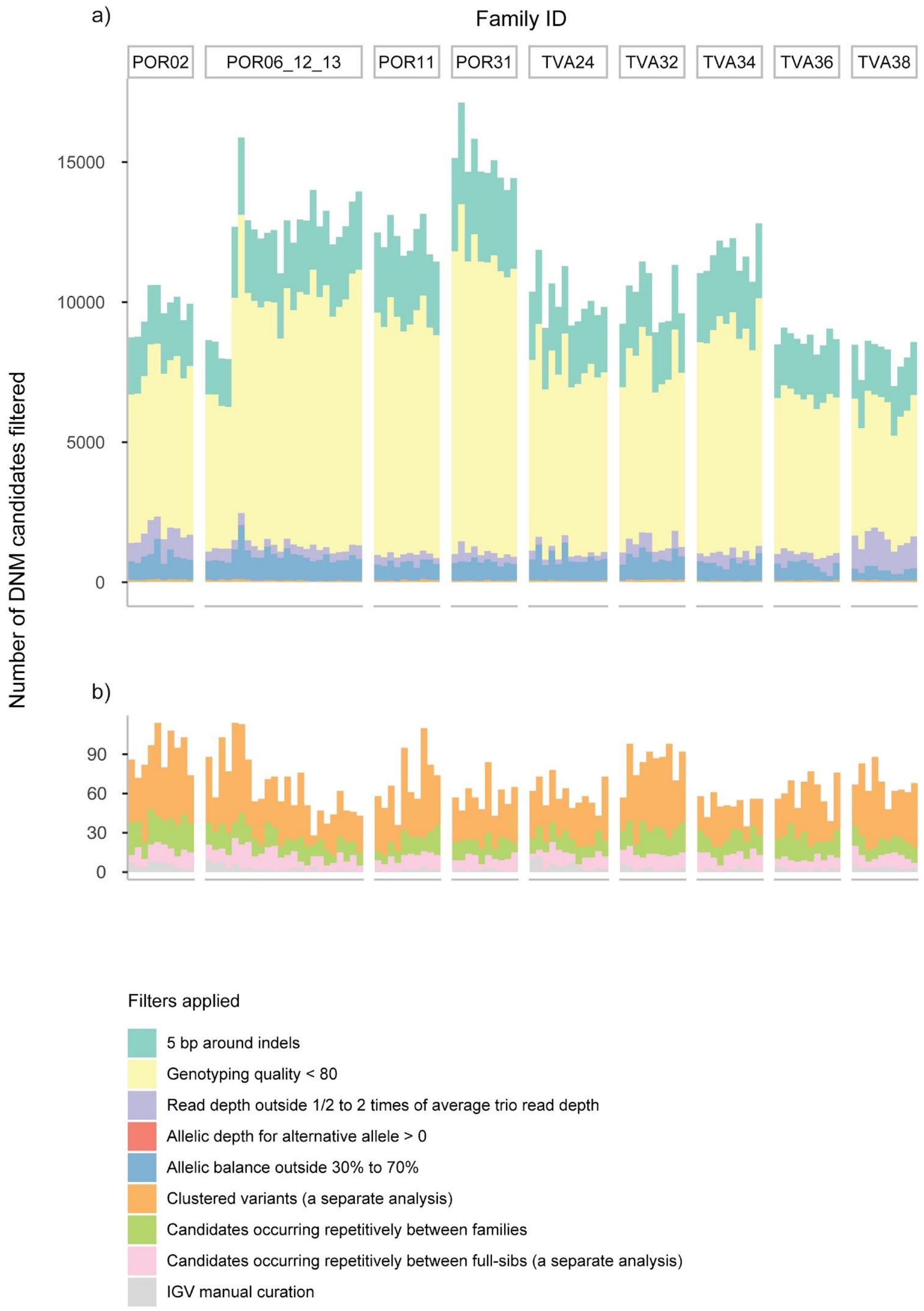

Supplementary Fig. 3. Comparison of DNM and transmission rates. There is no statistically significant difference in DNM rates between a) generations, b) sexes, or c) pedigree types. d) Transmission rates of the DNMs from F_1 to F_2 generation do not differ between inbred and outbred pedigrees.

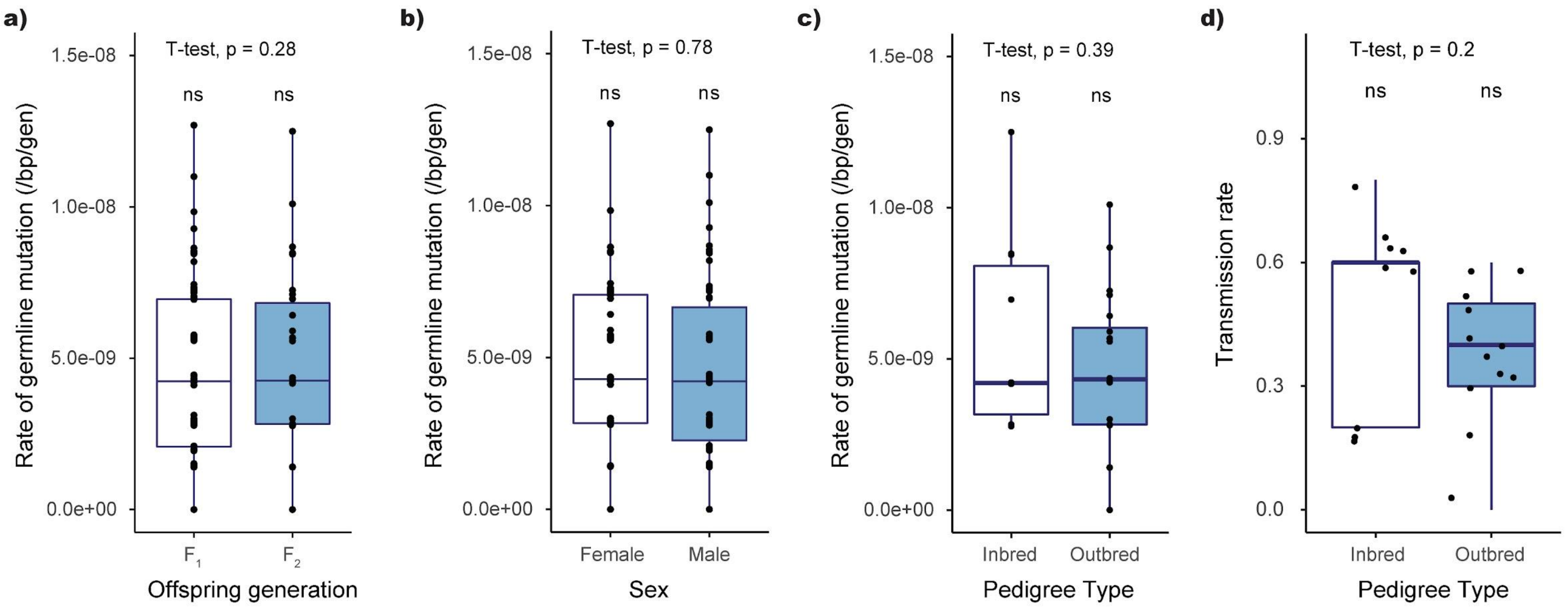

Supplementary Fig. 4. Detailed DNM- (above) and substitution- (below) rate based phylogenies. Node A: Divergence time (Mya) between *P. pungitius* and *G. aculeatus.* Node B: Divergence time among different *P. pungitius* lineages. Node C: Divergence time between western and eastern European *P. pungitius* lineages. The 95% HPDs of node ages are indicated by the blue bars at nodes. Nodes without white circles are supported by higher than 0.95 posterior values.

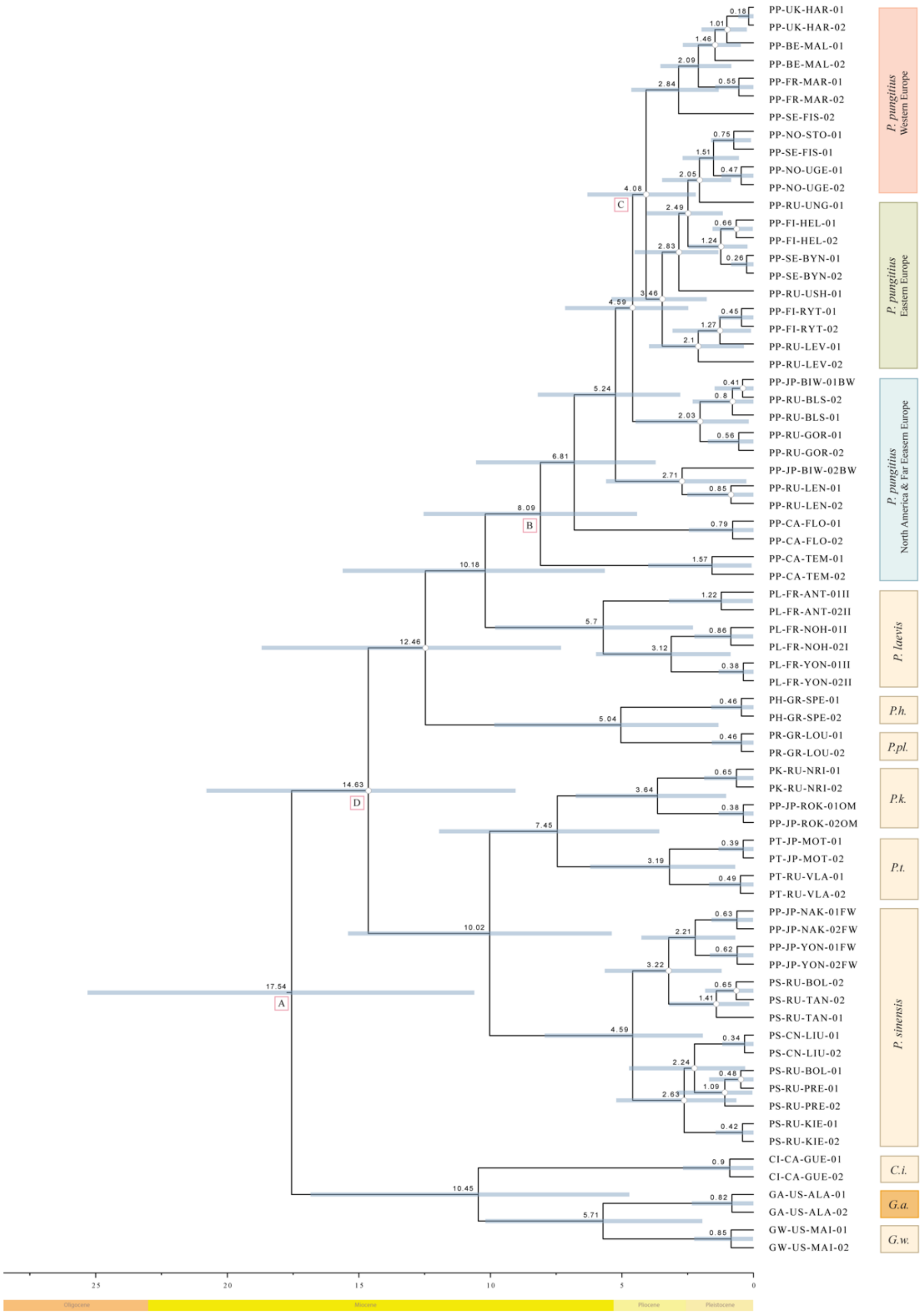

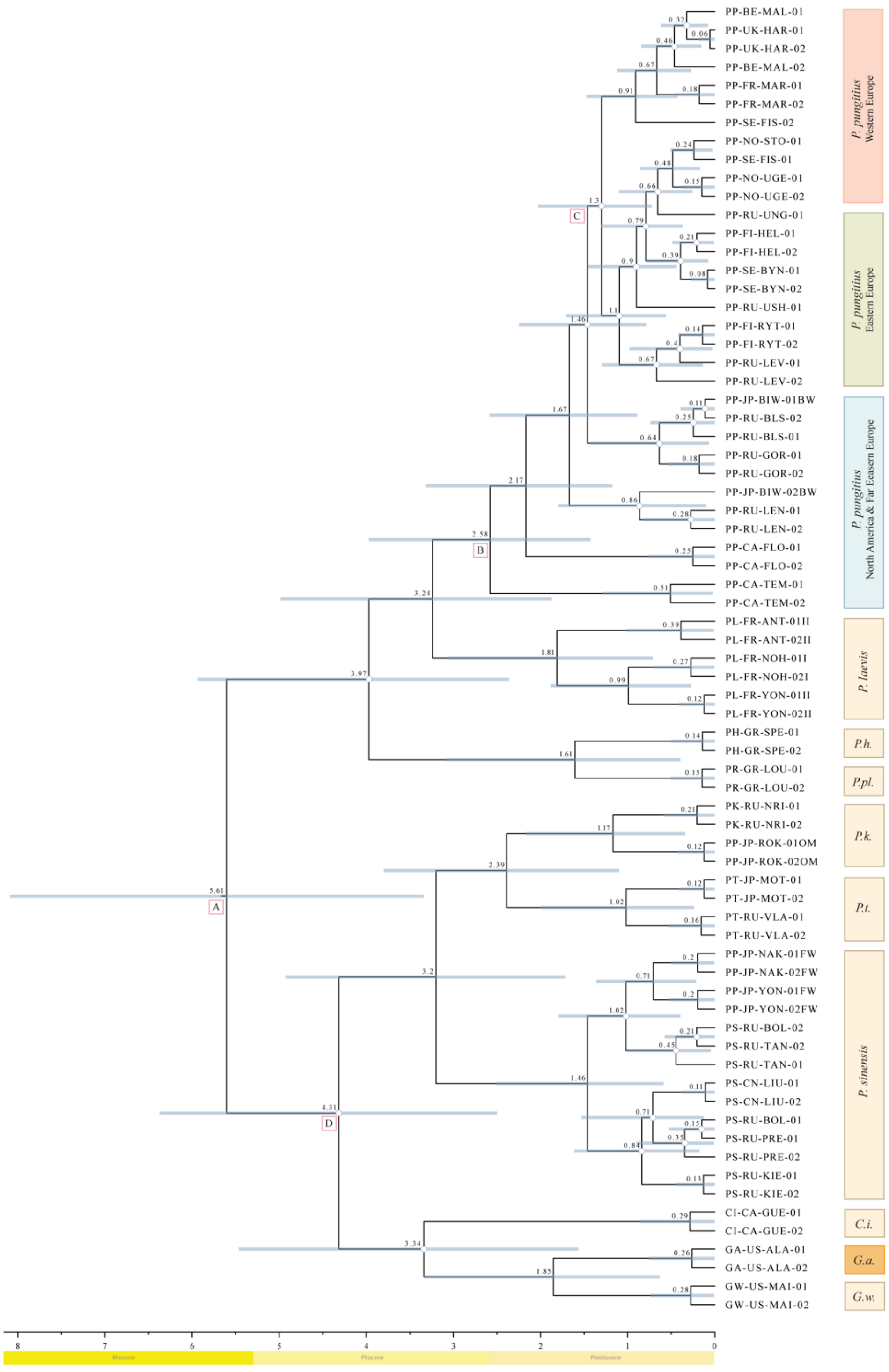

PP-BE-MAL-01
PP-UK-HAR-01
PP-UK-HAR-02
PP-BE-MAL-02
PP-FR-MAR-01
PP-FR-MAR-02
PP-SE-FIS-02
PP-NO-STO-01
PP-SE-FIS-01
PP-NO-UGE-01
PP-NO-UGE-02
PP-RU-UNG-01
PP-FI-HEL-01
PP-FI-HEL-02
PP-SE-BYN-01
PP-SE-BYN-02
PP-RU-USH-01
PP-FI-RYT-01
PP-FI-RYT-02
PP-RU-LEV-01
PP-RU-LEV-02
PP-JP-BIW-01BW
PP-RU-BLS-02
PP-RU-BLS-01
PP-RU-GOR-01
PP-RU-GOR-02
PP-JP-BIW-02BW
PP-RU-LEN-01
PP-RU-LEN-02
PP-CA-FLO-01
PP-CA-FLO-02
PP-CA-TEM-01
PP-CA-TEM-02
PL-FR-ANT-01II
PL-FR-ANT-02II
PL-FR-NOH-01I
PL-FR-NOH-02I
PL-FR-YON-01II
PL-FR-YON-02II
PH-GR-SPE-01
PH-GR-SPE-02
PR-GR-LOU-01
PR-GR-LOU-02
PK-RU-NRI-01
PK-RU-NRI-02
PP-JP-ROK-01OM
PP-JP-ROK-02OM
PT-JP-MOT-01
PT-JP-MOT-02
PT-RU-VLA-01
PT-RU-VLA-02
PP-JP-NAK-01FW
PP-JP-NAK-02FW
PP-JP-YON-01FW
PP-JP-YON-02FW
PS-RU-BOL-02
PS-RU-TAN-02
PS-RU-TAN-01
PS-CN-LIU-01
PS-CN-LIU-02
PS-RU-BOL-01
PS-RU-PRE-01
PS-RU-PRE-02
PS-RU-KIE-01
PS-RU-KIE-02
CI-CA-GUE-01
CI-CA-GUE-02
GA-US-ALA-01
GA-US-ALA-02
GW-US-MAI-01
GW-US-MAI-02
P. pungitius Western Europe
P. pungitius Eastern Europe
P. pungitius North America & Far Eeasern Europe
P. laevis
P.h.
P.pl.
P.k.
P.t.
P. sinensis
C.i.
G.a.
G.w.
A
B
C
D
5.61
3.97
3.24
2.58
2.17
1.67
1.46
1.3
1.1
4.31
3.2
3.34
2.39
1.81
1.61
1.17
1.02
1.46
1.85
8
7
6
5
4
3
2
1
0
Miocene
Pliocene
Pleistocene

Supplementary Fig. 5. Mutation spectra for the 214 phased mutations.

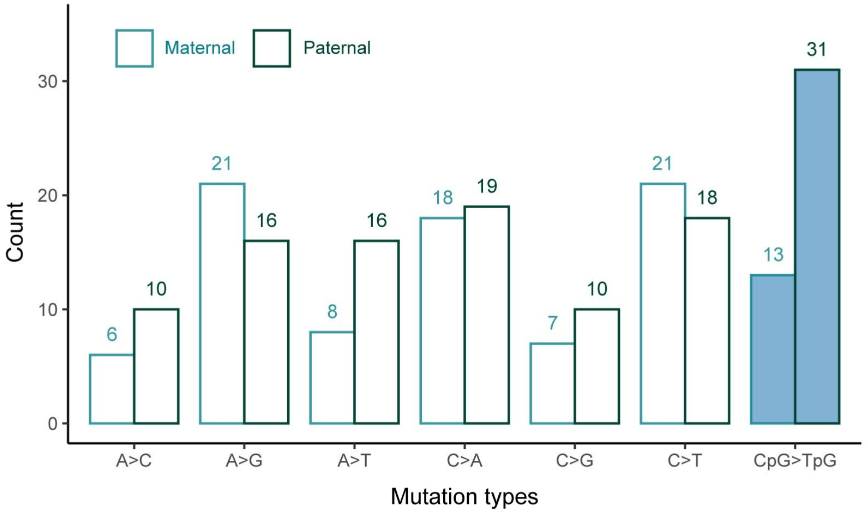

Supplementary Fig. 6. Number and rate of DNMs inside and outside CpG island (CGI). a) Number of CpG > TpG (blue) or other (white) DNMs detected at CGI (left) and non-CGI (right) regions. b) The rate of DNMs inside different types of CGI and non-CGI (TSS: transcription start site; TTS: transcription termination site).

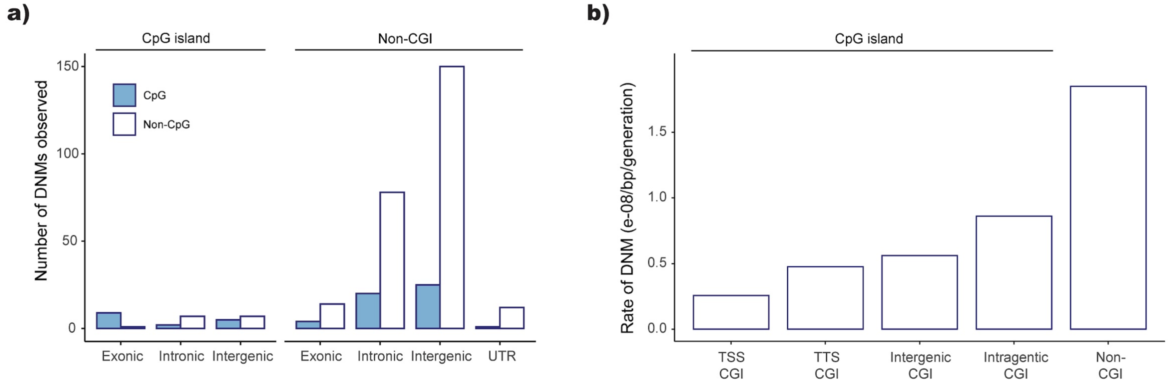

Supplementary table 1. DNM rates estimates from other pedigree-based studies.

**Species**	**DNM rates (E-08/gen/bp)**	**Number of trios**	**References**
Baboon (*Papio anubis*)	0.57*	12	Wu et al. 2020
Bolivian squirrel monkey (*Saimiri boliviensis boliviensis*)	0.69*	3	Bergeron et al. 2023
Chimpanzee (*Pan troglodytes*)	1.2	6	Venn et al. 2014

	1.48	1	Tatsumoto et al. 2017
	1.26	7	Besenbacher et al. 2019
	1.25	1	Bergeron et al. 2023
Drill (*Mandrillus leucophaeus*)	0.633*	2	Bergeron et al. 2023
Gorilla (*Gorilla gorilla*)	1.13	2	Besenbacher et al. 2019
Gray mouse lemur (*Microcebus murinus*)	1.52	2	Campbell et al. 2021
Green monkey (*Chlorocebus sabaeus*)	0.94	3	Pfeifer, 2017
Human (*Homo sapiens*)	1.17	1 (CEU)	Conrad et al. 2011
	0.97	1 (YRI)	
	1.2	78	Kong et al. 2012
	1.27	10	Besenbacher et al. 2015
	1.28	13	Rahbari et al. 2016
	1.05	719	Wong et al. 2016
	1.29	1550	Jónsson et al. 2017
	1.28	150	Maretty et al. 2017
	1.7	516	Turner et al. 2017
	1.1	593	Sasani et al. 2019
	1.22	1449	Kessler et al. 2020
	1.1665	2	Bergeron et al. 2023
Lar gibbon (*Hylobates lar*)	0.705	1	Bergeron et al. 2023
Marmoset (*Callithrix jacchus*)	0.43*	1	Yang et al. 2021
Orangutan (*Pongo abelii*)	1.66	1	Besenbacher et al. 2019
Owl monkey (*Aotus nancymaae*)	0.81	14	Thomas et al. 2018
Rhesus macaque (*Macaca mulatta*)	0.58*	14	Wang et al. 2020
	0.77	19	Bergeron et al. 2021
White-faced saki (*Pithecia pithecia*)	0.994	5	Bergeron et al. 2023
Alpaca (*Vicugna pacos*)	0.944	1	Bergeron et al. 2023
Antarctic fur seal (*Arctocephalus gazella*)	0.67825	4	

Bottlenose dolphin (*Tursiops truncatus*)	0.883	1	
Cape rock hyrax (*Procavia capensis*)	0.91966667	3	
Cattle (*Bos taurus*)	1.17	5	Harland et al. 2017
Cervus nippon (*Cervus nippon*)	0.471*	1	Bergeron et al. 2023
Chinese tree shrew (*Tupaia chinensis belangeri)*	0.75	3	
Damaran mole rat (*Fukomys damarensis*)	0.74075	4	
Dog (*Canis lupus familiaris*)	0.86825	4	
Domestic cat (*Felis catus*)	0.86	11	Wang et al. 2022
	0.349	1	Bergeron et al. 2023
Egyptian roussette (*Rousettus aegyptiacus*)	0.811	1	
Forest musk deer (*Moschus berezovskii*)	1	1	
Giraffe (*Giraffa camelopardalis*)	1.01	1	
Goat (*Capra hircus*)	0.526*	3	
Gray short-tailed opossum (*Monodelphis domestica*)	0.46*	1	
Guinea pig (*Cavia aperea*)	0.86683333	6	
Hippopotamus (*Hippopotamus amphibius*)	1.19	1	
Killer whale (*Orcinus orca*)	1.41	1	
Leopard (*Panthera pardus*)	0.884	1	
Malay tapir (*Tapirus indicus*)	0.48*	1	
Mouse (*Mus musculus*)	0.57	8	Milholland et al. 2017
	0.39*	15	Lindsay et al. 2019
	0.535	1	Bergeron et al. 2023
Neovison vison (*Neovison vison*)	0.464*	1	Bergeron et al. 2023
Pig (*Sus scrofa*)	0.36*	5	Zhang et al. 2022
	0.43185714*	7	Bergeron et al. 2023
Platypus (*Ornithorhynchus anatinus*)	0.7*	2	Martin et al. 2018
Red deer (*Cervus elaphus yarkandensis*)	0.671	1	Bergeron et al. 2023

Red fox (*Vulpes vulpes*)	0.446*	1	
Red panda (*Ailurus fulgens*)	1.31	2	
Reindeer (*Rangifer tarandus*)	0.5895*	2	
Southern White Rhinoceros (*Ceratotherium simum simum*)	0.956	1	
Tasmanian devil (*Sarcophilus harrisii*)	0.595	1	
Tiger (*Panthera tigris*)	0.699	1	
Walrus (*Odobenus rosmarus*)	1.32	1	
Wolf (*Canis lupus*)	0.45*	4	Koch et al. 2019
Atlantic salmon (*Salmo salar*)	0.431*	1	Bergeron et al. 2023
Cichlids (*Astatotilapia calliptera, Aulonocara stuartgranti,* and *Lethrinops lethrinus*)	0.35*	9	Malinsky et al. 2018
Common carp (*Cyprinus carpio*)	0.562	1	Bergeron et al. 2023
Common clownfish (*Amphiprion ocellaris*)	0.737*	2	
Gulf pipefish (*Syngnathus scovelli*)	0.558*	6	
Herring (*Clupea harengus*)	0.2	12	Feng et al. 2017
Japanese flounder (*Paralichthys olivaceus)*	0.8535	2	Bergeron et al. 2023
Large yellow croaker (*Larimichthys crocea*)	0.226	1	
Siamese fighting fish (*Betta splendens*)	0.49666667*	3	
Tongue sole (*Cynoglossus semilaevis*)	0.913	3	
Adelie penguin (*Pygoscelis adeliae*)	1.5	1	
Blackbird (*Turdus merula*)	0.697	4	
Blue tit (*Cyanistes caeruleus*)	0.51966667*	3	
Blue-throated macaw (*Ara glaucogularis*)	1	1	
Chicken (*Gallus gallus domesticus*)	0.363	1	
Collared flycatcher (*Ficedula albicollis*)	0.46*	7	Smeds et al. 2016
Dalmatian pelican (*Pelecanus crispus*)	0.912	1	Bergeron et al. 2023
Darwin's rhea (*Rhea pennata*)	3.98	1	

Emperor penguin (*Aptenodytes forsteri*)	1.41	1	
European herring gull (*Larus argentatus*)	0.74057143	7	
Great black-backed gull (*Larus marinus*)	0.873	2	
Greater Flamingo (*Phoenicopterus roseus*)	1.66	1	
Griffon vulture (*Gyps fulvus*)	0.261	1	
Quail (*Coturnix japonica*)	0.56725*	8	
Roseate spoonbill (*Platalea ajaja*)	1.69	1	
Siberian stonechat (*Saxicola maurus*)	0.6676	5	
Snowy owl (*Bubo scandiacus*)	0.098	1	
Southern screamer (*Chauna torquata*)	0.706	1	
Zebra finch (*Taeniopygia guttata*)	0.585*	2	
Common fruit fly (*Drosophila melanogaster*)	0.28*	12	Keightley et al. 2014
Fruit fly (*Drosophila simulans*)	0.451*	30	Wang et al. 2023
Butterfly (*Heliconius melpomene*)	0.29*	30	Keightley et al. 2015
Big-scaled least gecko (*Sphaerodactylus inigoi*)	1.36	1	Bergeron et al. 2023
Central bearded dragon (*Pogona vitticeps*)	1.43366667	3	
Common garter snake (*Thamnophis sirtalis*)	2.54333333	3	
Leopard gecko (*Eublepharis macularius*)	0.575	1	
Painted turtle (*Chrysemys picta*)	0.46077778*	9	
Texas banded gecko (*Coleonyx brevis*)	0.633	1	

Supplementary table 2. The number of DNM candidates left and the percentage being removed after each filter. For description of filters, see Methods. (RM: removed)

	Mendelian violation	Indel-5bp	GQ-80	DP-trio	AD-0	AB-0.3-0.7	RM-Clustered variants	RM-Repeated among pedigrees	RM-Repeated among full-sibs	DNM (not shared)
**Left after filtering**	11,006.9	8,596.9	1,256.6	789.3	787.5	69.0	30.1	15.4	5.0	2.2
**Percentage filtered**		21.90%	85.38%	37.19%	0.23%	91.24%	56.38%	48.84%	67.53%	56.00%

Separate check of the variants	Clustered variants	DNM (clustered)	Repeated between full-sibs	DNM (shared)
**Left after filtering**	38.9	0.1	10.4	0.8
**Percentage filtered**		99.67%		92.31%

	DNM (not shared)	DNM (clustered)	DNM (shared)	In total
**Average number of DNMs identified**	2.2	0.1	0.8	3.1
